# Metabolic Adaptations to Caloric Restriction: Time- and Group-Dependent Metabolomic Signatures from the CALERIE^™^ Trial

**DOI:** 10.21203/rs.3.rs-9237764/v1

**Published:** 2026-03-30

**Authors:** Melissa C. Orenduff, Emily K. Woolf, Ruiqi Zhang, Daniel W. Belsky, Sai Krupa Das, Waylon Hastings, Justine M. Mucinski, Susan B. Racette, Leanne M. Redman, Reem Waziry, Kari Wong, William E. Kraus, Carl F. Pieper, Kim M. Huffman

**Affiliations:** 1 Duke Molecular Physiology Institute, Duke University School of Medicine, Durham, NC, USA; 2 Department of Medicine, Duke University School of Medicine, Durham, NC, USA; 3 Reproductive Endocrinology and Women’s Health Laboratory, Pennington Biomedical Research Center, Baton Rouge, LA.; 4 Robert N Butler Columbia Aging Center, Columbia University Mailman School of Public Health, New York, NY, USA; 5 Department of Epidemiology, Columbia University Mailman School of Public Health, New York, NY, USA; 6 Jean Mayer USDA Human Nutrition Research Center on Aging at Tufts University, Boston, MA, USA; 7 Department of Nutrition, Texas A&M University, College Station, TX, USA; 8 Institute for Advancing Health through Agriculture, Texas A&M AgriLife Research, College Station, TX, USA; 9 AdventHealth Translational Research Institute, AdventHealth Orlando, Orlando, FL, USA.; 10 College of Health Solutions, Arizona State University, Phoenix, AZ, USA; 11 Department of Neurology, Vagelos College of Physicians and Surgeons, Columbia University, New York, USA; 12 Metabolon, Inc. Morrisville, North Carolina, USA; 13 Center on Aging and Development, Biostatistics and Bioinformatics, Duke University, Durham, NC, USA

**Keywords:** CALERIE^™^, Caloric Restriction, Metabolomics, principal component analysis (PCA)

## Abstract

**Background:**

Caloric restriction (CR) improves markers of biological aging, yet long-term effects on the human metabolome remain unclear.

**Objective:**

This study examined the effects of CR (2 years) in healthy adults without obesity on circulating metabolites linked to aging and metabolic adaptations.

**Methods:**

Untargeted metabolomics was performed using fasted plasma samples collected at baseline, 12, and 24 months (BL, 12M, 24M) from CALERIE^™^ participants randomized to CR or *ad libitum* (AL) control. A total of 864 known metabolites were identified and grouped into nine biologically coherent super pathways to support pathway-level interpretation (amino acid, peptide, carbohydrate, energy, lipid, nucleotide, cofactors and vitamins, xenobiotics, and partially characterized molecules). Principal component analysis (PCA) summarized metabolite variation, and linear mixed models assessed intervention effects on each PC in group-by-time interactions.

**Results:**

Three principal components showed significant group-by-time interactions: PC2 (carbohydrate), PC5 (partially characterized molecules), and PC4 (lipid). Carbohydrate (PC2) and partially characterized metabolites (PC5) decreased from baseline to 12M in both groups; from 12M to 24M, levels stabilized in CR but increased in AL for PC2, while PC5 continued to decline in AL and increased in CR. Lipid metabolites (PC4) decreased in CR and increased in AL at 12M, with the pattern reversing from 12M to 24M. Key contributors included malto-saccharides and related carbohydrate intermediates for PC2, glutamine degradants and lactone sulfates for PC5, and sphingolipids for PC4.

**Conclusion:**

Calorie restriction produced distinct, time-dependent shifts in carbohydrate and lipid metabolism, with early reductions during the weight-loss phase followed by stabilization or compensatory responses during weight maintenance. These dynamic metabolic changes may relate to inflammation-linked mechanisms. Further work is needed to distinguish CR-specific adaptations from dietary influences and to clarify the functional significance of these metabolic responses for aging and long-term metabolic health.

## INTRODUCTION

The Comprehensive Assessment of Long-Term Effects of Reducing Intake of Energy (CALERIE^™^) study is a landmark investigation into the effects of caloric restriction (CR) on human health and aging. The study aimed to determine the effects of long-term (2 years) CR among healthy adults without obesity compared with an *ad libitum* (AL) control condition. Participants who underwent CR achieved an average of 11.9% caloric reduction and sustained a 10% weight loss over two years (1). Post-hoc molecular analyses suggest modest CR can activate biological pathways associated with healthy aging and potentially delay the onset of age-related diseases (2). Metabolism is a key component in understanding how CR influences health and aging. Previous preclinical studies have demonstrated that CR can lead to significant changes in metabolic processes, including improved insulin sensitivity, reduced inflammation, and enhanced mitochondrial function (3–5). These metabolic adaptations are thought to contribute to observed lifespan extension and delayed onset of age-related diseases observed in animal models; however, the effects of long-term CR on metabolites and biochemical activities within the ‘healthy’ human metabolome are not well understood.

To address this gap, we performed a global, untargeted metabolomics analysis on circulating metabolites to investigate changes and pathways influenced by CR, which could provide insights into the metabolic mechanisms underlying health benefits and potential impacts on healthy aging.

## METHODS

### CALERIE^™^ Study Design and Intervention

The design and methods of CALERIE^™^ have been described in detail elsewhere (6, 7). Briefly, participants were early- to mid-life adults (men 20–50 years; women 20–47 years) with body mass index values in the normal-weight to overweight range (22.0 to <28.0 kg/m^2^). Participants (n=220) were randomized 2:1 to the CR intervention (n=145) or *ad libitum* (AL) control (n=75) groups (6, 7). Of the 220 participants randomized, 183 individuals (CR = 115; AL = 68) provided plasma samples at all three timepoints [(baseline (BL), 12 months (12M), and 24 months (24M)] and were included in the final analysis. All study participants provided written informed consent, and the study protocol was approved by the Institutional Review Boards at each of the three clinical sites (Washington University School of Medicine, St Louis, MO, USA; Pennington Biomedical Research Center, Baton Rouge, LA, USA; Tufts University, Boston, MA, USA). Duke University (Durham, NC, USA) served as the coordinating site. Phenotypic and clinical data were obtained from the CALERIE^™^ Biorepository (https://calerie.duke.edu/apply-samples-and-data-analysis). CALERIE^™^ Phase 2 clinical trial was approved by the Institutional Review Board (IRB:00006471) and registered with clinical trials.gov (NCT00427193) on January 2007.

### Sample Collection and Metabolomic Profiling.

Plasma was isolated by centrifugation of blood samples collected in EDTA tubes after an overnight fast (12 h) at three timepoints: BL, 12M, and 24M. Samples were processed and stored at −80°C for analyses at Metabolon^®^ (Durham, NC, USA). Metabolite concentrations were measured using an untargeted Ultrahigh Performance Liquid Chromatography-Tandem Mass Spectroscopy (UPLC-MS/MS) and quantified using the area under the curve of primary MS ions. Of the 1118 untargeted compounds measured, n=864 metabolites had a known identity using Metabolon’s web-service software.

### Metabolite Preprocessing.

Prior to analysis, metabolites with known identities were normalized to sample volume (each metabolite value divided by the corresponding sample’s volume) and then batch-normalized. Missing values, when present, were imputed using the minimum observed value for each compound.

### Super Pathway Grouping.

Metabolites with known identities (n = 1,118) were grouped into nine predefined super pathways to reduce feature complexity and facilitate pathway-level interpretation. These super pathways -- **amino acids**, **peptides**, **carbohydrates**, **energy**, **lipids**, **nucleotides**, **cofactors and vitamins**, **xenobiotics**, and **partially characterized molecules** -- follow Metabolon^®^, Inc.’s biochemical function–based classification.

### Principal Component Analysis (PCA) Within Super Pathways.

For PCA, data were centered and scaled (i.e., each variable was mean-centered and divided by its standard deviation to achieve unit variance). For each super pathway *SP*_*j*_, Principal Component Analysis (PCA) was applied to baseline-normalized metabolite concentrations to summarize shared variance among correlated metabolites. Principal components were retained using the elbow criterion, which identifies the point on the scree plot where additional components contribute minimal incremental variance (8, 9). This procedure yielded *X*_*j*_ retained components for each super pathway (**Supplemental Table 1**), providing a reduced set of pathway-level features for downstream modeling.

### Computation of Participant-Level PCA Scores.

To assess change over time, metabolite concentrations for each participant *i ∈ N* and metabolite *p ∈ P* were normalized at 12M and 24M using baseline statistics:

$$Z_{ipt}=\frac{X_{ipt}-{\bar{X}}_{p}}{S_{p}},$$

where *X*_*ipt*_is the concentration of metabolite *p* for participant *i* at time t, ${\bar{X}}_{p}$ is the mean baseline concentration of metabolite *p*, and *S*_*p*_ is the corresponding baseline standard deviation.

Using these normalized values, principal component scores for each retained component *PC*_*y*_ were computed for each participant and timepoint as:

$$PC_{y,it}=\sum_{p}F_{y,p}\cdot Z_{ipt},$$

where *F*_*y,p*_ denotes the loading coefficient of metabolite *p* on component *PC*_*y*_. The resulting time-specific component scores were then used as longitudinal outcomes in mixed-effects models to evaluate the effects of caloric restriction over time.

### Mixed-Model Longitudinal Analyses.

To evaluate the effects of group and time on metabolite variation, linear mixed-effects models were implemented in R (version 4.4.0). For each principal component *PC*_*y*_, the following repeated-measures model was estimated:

$$PC_{y,it}=\beta_{0}+\beta_{1}\left(\text{Baseline}\;{\text{PC}}_{y,i}\right)+\beta_{2}\left({\text{Timepoint}}_{t}\right)+\beta_{3}\left({\text{Sex}}_{i}\right)+\beta_{4}\left({\text{Group}}_{i}\right)+\beta_{5}\left({\text{Site}}_{i}\right)+\beta_{6}\left(\text{Baseline}\;{\text{BMI}}_{i}\right)+\beta_{7}\left({\text{Timepoint}}_{t}\times {\text{Group}}_{i}\right)+u_{i}+\epsilon_{it},$$

where Timepoint was coded as 0 for 12M and 1 for 24M; Group as 1 for CR and 0 for AL; and Sex as 0 for female and 1 for male. The random intercept *u*_*i*_ accounted for within-participant correlation across repeated measures.

The intercept *β*_*0*_ represents the expected value of *PC*_*y*_ at 12M among females in the AL group, controlling for baseline PC score, study site, and baseline BMI. The interaction term *β*_*7*_ tests whether the effect of caloric restriction differs between 12M and 24M. Sex, site, and baseline BMI were included as covariates but were not interacted with time or group. Given the exploratory nature of the analysis, no adjustments were made for multiple comparisons. Results are presented in tabular format, with statistical significance defined as *P* < 0.05.

## RESULTS

### Participant Baseline Characteristics

Study participants (*n* = 183; CR: *n* = 115, AL: *n* = 68) were predominantly female (69%), approximately 38 years old, and White (>70%), with a mean BMI of 25.2 kg/m^2^ (Table 1). Although the CR group was prescribed a 25% reduction in caloric intake, the AL group also exhibited slight decreases. Relative to baseline, mean caloric intake declined by 15% in the CR group and 1% in the AL group at 12M, and by 12% and 0.6%, respectively, at 24M (Table 2). Body weight in the CR group decreased by 11.7 ± 3.8 kg from baseline to 12M and remained stable through 24M (Table 2).

### CR versus AL trajectories of metabolite components over 12 and 24 months

**Figure 1.**
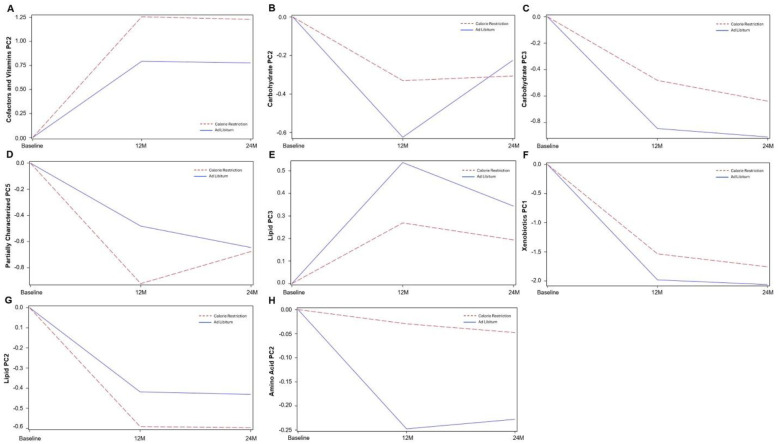
Group-specific differences in metabolite component trajectories over 12M and 24M. Model-estimated trajectories of principal component (PC) scores for metabolite groups that differed between CR (red-dashed lines) and AL (blue solid line) arms over 12M and 24M. Lines represent predicted mean values from mixed-effects models adjusted for baseline metabolite levels, BMI, sex, and site. Statistical estimates and +/−SEs are provided in Supplemental Table 2.

Across multiple metabolite pathways, CR and AL showed distinct PC trajectories over 12M and 24M (see Figure 1A–H). Cofactors and Vitamins (PC2) increased from BL to 12M in both groups (CR and AL) and then plateaued at 24M (0.46 ± 0.12; p=0.0002). Carbohydrate (PC2) decreased from BL to 12M in both arms, followed by a plateau in CR and a rebound in AL from 12M to 24M (0.29 ± 0.08; p=0.0003). Carbohydrate (PC3) decreased steadily across both intervals (BL–12M and 12M–24M) in both groups (0.36 ± 0.11; p=0.001).

For Partially Characterized Molecules (PC5), both CR and AL decreased from BL to 12M; however, CR rebounded while AL continued to decline 12M to 24M (–0.44 ± 0.14; p=0.002). Lipid (PC3) increased at 12M and declined at 24M in both groups (–0.27 ± 0.10; p=0.01). Xenobiotics (PC1) decreased in both arms, at both follow-up timepoints (12M and 24M) (0.45 ± 0.18; p=0.02). Lipid (PC2) declined from BL to 12M and then plateaued at 24M in both groups (–0.17 ± 0.08; p=0.03). Amino Acid (PC2) remained relatively stable in CR, whereas AL showed a marked decline at 12M followed by partial recovery at 24M (0.22 ± 0.11; p=0.04). Statistical estimates and standard errors are reported in **Supplemental Table 2**.

### Time-Dependent Changes in Metabolite Abundance in CR and AL Groups

**Figure 2.**
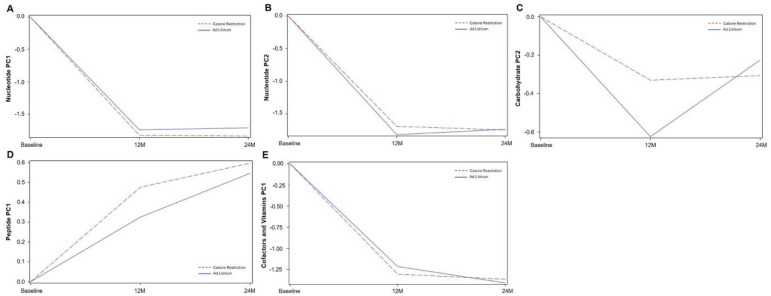
Time-dependent trajectories of metabolite components in CR and AL groups. Model-estimated trajectories of principal component (PC) scores for metabolite groups exhibiting time-dependent changes between BL, 12M, and 24M across groups [CR (red-dashed lines) / AL (blue solid line)]. Lines represent predicted mean values from mixed-effects models adjusted for baseline metabolite levels, BMI, sex, and site. Statistical estimates and +/−SEs are provided in Supplemental Table 2.

Across several metabolite pathways, CR and AL exhibited broadly similar temporal patterns (see Figure 2A–E). Nucleotide (PC1) declined sharply from BL to 12M in both groups and then stabilized at 24M (−1.74 ± 0.72; p=0.02). Nucleotide (PC2) showed a nearly identical pattern, with a marked reduction at 12M followed by minimal change at 24M(−1.82 ± 0.77; p=0.02). Carbohydrate (PC2) decreased from BL to 12M in both arms, followed by partial recovery at 24 months (0.40 ± 0.087; p=0.000006). Peptide (PC1) increased steadily at 12M and 24M in both groups (0.22 ± 0.098; p=0.024). Cofactors and Vitamins (PC1) declined substantially from BL to 12M in both arms and then remained stable at 24M (−0.19 ± 0.097; p=0.047). Statistical estimates and standard errors for all components are provided in **Supplemental Table 2**.

### CR-by-time differences in super-pathway components

**Figure 3.**
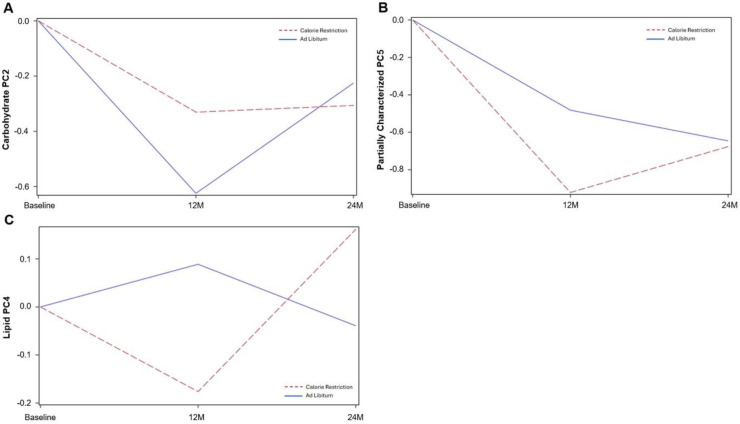
CR Group by time differences in Super pathway PCs. Model-estimated trajectories of principal component (PC) scores for metabolite groups demonstrating significant time × group interactions over 12M and 24M. CR (red-dashed lines) and AL (blue solid line). Lines represent predicted mean values from mixed-effects models adjusted for baseline metabolite levels, BMI, sex, and site. Statistical estimates and +/−SEs are provided in Supplemental Table 2.

Significant time × group interactions were observed for Carbohydrate (PC2), Partially Characterized Molecules (PC5), and Lipid (PC4) (see Figure 3A–C). For Carbohydrate (PC2), the magnitude of the CR–AL difference decreased by 0.37 ± 0.11 units at 24M relative to 12M (P=0.0007); both groups declined from BL to 12M, but from 12M to 24M, levels plateaued in CR while increasing in AL. For Partially Characterized Molecules (PC5), the magnitude of the group difference increased by 0.41 ± 0.15 units at 24M compared with 12M (P=0.009); both groups declined initially, but CR rebounded from 12M to 24M while AL continued to decline. Lipid (PC4) demonstrated distinct mid-study patterns, with AL showing a modest rise at 12M followed by a decline at 24M, while CR decreased at 12M and then rebounded by 24M. The magnitude of the CR–AL difference at 24M was 0.47 ± 0.23 units greater than at 12M (P=0.04). Statistical estimates and standard errors are provided in **Supplemental Table 2**.

### Principal Component Constituents

Metabolite contributions with the greatest loadings (>2%) on each principal component associated with group, time, and time × group effects are listed in **Supplemental Table 3**.

## DISCUSSION

This study provides the first report of how long-term CR influences circulating metabolic intermediates over a two-year randomized controlled trial in healthy adults without obesity. Using PCA-derived super-pathway metabolite components and longitudinal mixed-effects models, we identified metabolite patterns that changed with CR and differed from AL, revealing both metabolic responses during active weight loss (BL to 12M) and later adaptations during weight maintenance (12M to 24M). These findings highlight metabolic pathways through which CR may exert cardiometabolic and anti-inflammatory effects.

### Carbohydrate Metabolism (Carbohydrate PC2)

Carbohydrate PC2 included metabolites such as maltotriose, maltose, maltotetraose, X3-phosphoglycerate, xylose, arabinose, and N-acetylneuraminate -- metabolites spanning glycolysis, glycogen breakdown, pentose metabolism, and immune-related glycan biology. Maltose- and maltotriose-containing oligosaccharides are classical products of α-amylase–mediated starch degradation (10–12). X3-phosphoglycerate is a well-established intermediate in glycolysis and gluconeogenesis and reflects central energy metabolism (13–15), while xylose and arabinose are pentose sugars typically derived from the breakdown of plant-based polysaccharides, including dietary fibers (13, 16, 17); their circulating levels may be influenced by dietary intake or gut microbial metabolism (18, 19), although the specific source cannot be determined from plasma metabolomics alone. N-acetylneuraminate (sialic acid) is a terminal glycan with established roles in immune activation, leukocyte signaling, and inflammatory responses (20–22).

Both CR and AL groups showed reductions in these metabolites from BL to 12M, but the rebound from 12M to 24M was markedly attenuated in CR. These patterns likely reflect the initial reduction in energy intake during the first year of CR, followed by longer-term physiological adjustments as participants transitioned to weight maintenance. Such adaptations are consistent with prior evidence that sustained caloric restriction induces coordinated metabolic and physiological changes, including reductions in energy expenditure and shifts in metabolic processes that enhance metabolic efficiency and reduce oxidative stress (23). Reduced availability of carbohydrate precursors (e.g., starch-derived saccharides) may also contribute to the observed declines. Notably, sustained reductions in N-acetylneuraminate with CR may reflect anti-inflammatory adaptations, given the central role of sialic acids in immune activation and inflammatory signaling (20, 21). Although AL participants exhibited a rebound in these metabolites, the stabilization in CR suggests more durable remodeling of carbohydrate and glycan-linked pathways during long-term CR

### Lipid Metabolism (Lipid PC4)

Lipid PC4 was dominated by sphingolipids, including sphinganine, sphingosine, and their phosphorylated forms -- bioactive lipids central to membrane organization, cell signaling, and inflammatory regulation (24–26). CR produced a sharp decline in these metabolites at 12M, followed by an increase at 24M to levels exceeding those of AL. AL showed a different pattern, with a modest rise at 12M followed by a decline at 24M. The early decline with CR may reflect acute reductions in inflammatory tone and shifts in sphingolipid-mediated signaling during active weight loss, consistent with known CR-associated improvements in inflammatory lipid profiles (23, 27). The subsequent rise from 12M to 24M may represent longer-term metabolic adaptation as sphingolipid pools re-equilibrate under sustained CR, aligning with evidence that sphingolipid metabolism responds dynamically to changes in energetic state (28, 29). Preclinical studies similarly report CR-induced increases in sphinganine species, interpreted as adaptive remodeling of lipid signaling pathways (30, 31). Thus, the biphasic pattern observed here may reflect coordinated, phase-specific remodeling of sphingolipid biology across the transition from active weight loss to long-term CR maintenance.

### Partially Characterized Molecules (PC5)

PC5 included glutamine degradants, pentose acids, branched-chain fatty acids, metabolonic lactone sulfates, and glycine conjugates -- metabolite classes linked to amino acid turnover, carbohydrate oxidation, lipid remodeling, and detoxification pathways. CR produced larger reductions at 12M than AL, followed by partial rebound at 24M, whereas AL showed progressive declines across both timepoints. Metabolonic lactone sulfate, a xenobiotic-related metabolite associated with adiposity and cardiometabolic risk (32), declined substantially with CR during active weight loss, consistent with improvements in metabolic health. Glutamine degradants and pentose acids may reflect shifts in amino acid metabolism and reduced inflammatory activity, given that glutamine serves as a key fuel for proliferating immune and tumor cells (33, 34). Reductions in glycine conjugates may indicate lower detoxification demand or changes in dietary patterns toward less processed foods, as glycine conjugation is a major pathway for clearance of xenobiotics and lipid-derived acids (35). Because many of these metabolites remain partially characterized, their biological roles require further investigation. Nonetheless, the observed patterns suggest that CR influences multiple interconnected metabolic pathways related to inflammation, amino acid turnover, and detoxification, consistent with broader evidence that CR induces coordinated remodeling across metabolic networks (23, 36, 37).

### Strengths

The randomized controlled design with a well-defined *ad libitum* comparator provides a rigorous framework for isolating the metabolic effects of sustained CR. The two-year intervention with fasting plasma collected at BL, 12M, and 24M offers rare longitudinal resolution to distinguish weight-loss–related changes at 12M from later weight-maintenance adaptations at 24M. Use of a comprehensive untargeted metabolomics platform (864 metabolites across nine super pathways), combined with principal component analysis and mixed-effects modeling, enabled systems-level characterization of CR-related metabolic remodeling while accounting for within-person change. The focus on healthy adults without obesity minimized confounding by metabolic disease, strengthening inference about CR-specific physiological adaptations. Together, these features make this one of the most detailed longitudinal metabolomic evaluations of CR in humans.

### Limitations

The cohort’s demographic homogeneity (predominantly White, healthy, non-obese adults) limits generalizability. Untargeted metabolomics provides relative rather than absolute quantification, and many partially characterized metabolites have uncertain identities or functions, constraining mechanistic interpretation. PCA aggregates metabolites across pathways, potentially reducing biological specificity. Dietary composition was not controlled, making it difficult to separate CR-specific effects from changes in food quality or macronutrient intake, and early weight-loss effects may overlap with longer-term CR mechanisms. Finally, the design did not capture acute metabolic responses occurring more acutely after initiating CR, which may contribute additional insight into early metabolic dynamics.

## CONCLUSION

Sustained caloric restriction drives coordinated, time-dependent remodeling across multiple metabolic pathways, with early shifts reflecting active weight loss and later patterns indicating durable physiological adaptation. Across diverse metabolite classes, CR produced consistent divergence from AL intake, revealing a broad reorganization of metabolic networks rather than isolated pathway effects. These findings position CR as a potent modulator of systemic metabolism in healthy adults and underscore the value of longitudinal metabolomics for defining the temporal architecture of human metabolic adaptation.

## Supplementary Material

Supplementary Files

This is a list of supplementary files associated with this preprint. Click to download.

- SupplementalTables123CALERIEmetabolomicsmanuscript.docx

## Figures and Tables

**Table 1.** Baseline characteristics for CALERIE^™^ 2 participants.

	Calorie Restriction (N=115)	*Ab libitum* (N=68)
**Sex (n(%))**		
Female	79 (68.7%)	47 (69.1%)
Male	36 (31.3%)	21 (20.9%)
**Age (y)**		
Mean (SD)	38.6 (7.3)	38.2 (7.0)
Median [min, max)	40.8 [21.3, 50.9]	38.7 [23.2, 50.9]
**Race**		
Asian	7 (6.1%)	3 (4.4%)
Black	0 (0%)	0 (0%)
White	91 (79.1%)	50 (73.5%)
Other	5 (4.3%)	4 (5.9%)
Missing	12 (10.4%)	11 (16.2%)
**Body Mass Index (kg/m** ^**2**^ **)**		
Mean (SD)	25.2 (1.7)	25.2 (1.6)
Median [min, max]	25.1 [21.3, 29.0]	24.9 [21.5, 28.4]

**Table 2.** Participant characteristics at 12 and 24 months relative to baseline for CALERIE^™^ 2 participants.

	12 months		24 months	
	Calorie Restriction (N=115)	*Ab libitum* (N=68)	Calorie Restriction (N=115)	*Ab libitum* (N=68)
Calorie	15.10 (7.21)	1.03 (9.08)	11.60 (7.09)	0.63 (8.19)
Restriction (%)				
Fat Free Mass (kg)	46.90 (8.97)	47.90 (8.59)	46.80 (9.18)	48.0 (8.38)
Δ Fat Free Mass	−2.05 (1.37)	−0.10 (1.11)	−2.01 (1.77)	−0.01 (1.65)
Fat Mass (kg)	17.30 (3.97)	23.70 (5.95)	18.10 (4.17)	24.30 (5.91)
Δ Fat Mass	−6.25 (2.49)	−0.26 (2.38)	−5.41 (2.72)	0.39 (3.34)
%Δ Weight	−11.70 (3.83)	−0.69 (4.20)	−10.40 (4.48)	0.42 (6.01)

Data are presented as mean (SD).

## Data Availability

Phenotypic and clinical data publicly available and accessible from the CALERIE^™^ Biorepository (https://calerie.duke.edu/apply-samples-and-data-analysis). De-identified human/patient standardized data type have been deposited at the Aging Research Biobank (https://agingresearchbiobank.nia.nih.gov/studies/calerie/) and are available upon request. To request access, please complete the survey and submit request at https://agingresearchbiobank.nia.nih.gov/how-to-make-a-request/. Summary statistics describing these data/processed datasets are deposited at https://calerie.duke.edu/apply-samples-and-data-analysis and are publicly available as of the date of publication.
